# Evaluation of the Potential of a Fast-Curing Polymer Bioadhesive Hydrogel for Corneal Defect Repair

**DOI:** 10.3390/gels12050357

**Published:** 2026-04-23

**Authors:** Zohreh Arabpour, Soheil Sojdeh, Amirhosein Panjipour, Zahra Bibak Bejandi, Amal Yaghmour, Miranda Castillo, Anwar N. Khandaker, Mohammad Soleimani, Ali R. Djalilian

**Affiliations:** 1 Department of Ophthalmology and Visual Science, University of Illinois, Chicago, IL 60612, USA; sojdesoheil@gmail.com (S.S.);; 2 Department of Ophthalmology, School of Medicine, University of North Carolina at Chapel Hill, Chapel Hill, NC 27599, USA

**Keywords:** corneal defect, fast-curable polymer, corneal glue, bioadhesive hydrogel, tissue adhesion, ionic crosslinking, ex vivo corneal sealing

## Abstract

Corneal defects are a major cause of vision loss and require rapid, biocompatible, and effective sealing methods to restore ocular integrity and prevent infection. Current clinical adhesives, such as cyanoacrylate and fibrin glue, are limited by problems such as poor biocompatibility and inadequate stability. This study presents the design and evaluation of a fast-curable polymer bioadhesive hydrogel, a corneal glue formulated for efficient sealing of corneal defects. Hydrogels were synthesized from natural and synthetic polymers, including polyvinyl alcohol (PVA), sodium alginate (SA), and carboxymethyl cellulose (CMC), optimized for rapid gelation (~45 s), robust adhesion (~15 kPa), and mechanical strength (tensile strength ~0.35 MPa and storage modulus G′ indicating strong elastic behavior). Physicochemical and rheological properties, including swelling behavior and optical transparency (>90% transmittance across 400–700 nm), were characterized, including gelation time, swelling behavior, and mechanical strength. In vitro biocompatibility was assessed using human corneal epithelial cells to evaluate cytotoxicity and cell adhesion. Ex vivo studies on human cadaveric corneas with full-thickness defects measured adhesive strength and sealing efficacy through burst pressure (~38 mmHg) and leakage tests, with comparisons to commercial fibrin and cyanoacrylate adhesives. The optimized corneal glue exhibited fast curing, robust adhesion, high water retention with minimal swelling, favorable viscoelastic properties, and excellent cytocompatibility effectively sealing corneal defects in ex vivo models. These results highlight its potential as a promising fast-curable bioadhesive for corneal wound repair and ocular surface restoration.

## 1. Introduction

Corneal injuries and ulcerative disorders represent major global causes of visual impairment [1]. Despite the high demand for corneal transplants, only a small fraction of patients in need receive timely surgery, with global transplant wait times often exceeding 12 months due to severe shortages of donor tissue [2,3]. This urgent gap highlights the critical need for alternative, rapidly deployable therapies to restore corneal integrity. Prolonged corneal infections, inflammation, and scarring can lead to progressive stromal thinning, compromising the biomechanical integrity of the cornea and potentially resulting in full-thickness perforations [4]. Such perforations, whether caused by microbial infections, autoimmune diseases, or trauma, demand immediate intervention to prevent severe complications including endophthalmitis, glaucoma, and intraocular infection [5].

Due to the scarcity of donor tissue and the complexity of surgical grafting, bioadhesive materials have gained increasing attention as practical and effective alternatives for emergency corneal repair [6]. For over fifty years, polymer-based adhesives and sealants have been used in ophthalmic surgery, functioning by crosslinking at the wound site to form stable mechanical and chemical bonds between tissue layers. Compared with traditional suturing, bioadhesives offer distinct advantages minimizing inflammation, reducing operation time, improving postoperative comfort, and providing mechanical properties closer to those of native corneal tissue [6].

To function effectively, a corneal adhesive must exhibit high optical transparency, biocompatibility, flexibility, and strong adhesion under wet conditions [7]. However, currently available clinical adhesives do not fully meet these requirements. Cyanoacrylate glues, though fast-curing and strong, release heat during polymerization and can cause cytotoxicity and irregular surface morphology [8]. Fibrin glues, while more biocompatible and capable of supporting tissue healing, often show weak adhesion to moist ocular tissues and poor mechanical resilience [9]. PEG-based sealants such as ReSure^®^ and OcuSeal^®^ demonstrate good handling and safety profiles but are primarily designed for sealing surgical incisions rather than repairing stromal defects [10].

To address these challenges, hydrogel-based adhesives have recently emerged as a new class of biomaterials offering both structural reinforcement and biological compatibility. Hydrogels can be engineered to possess high water content, tunable stiffness, and tissue-like elasticity, providing a suitable microenvironment for cellular interaction and regeneration [11]. Hybrid hydrogels combining natural polymers such as alginate and carboxymethyl cellulose (CMC), combined with synthetic components like polyvinyl alcohol (PVA), leverage dual mechanisms: physical polymer entanglement and Ca^2+^-mediated ionic crosslinking. This design creates hybrid systems that achieve an optimal balance between strength, transparency, and biocompatibility. Furthermore, incorporating rapid crosslinking mechanisms allows these hydrogels to solidify quickly upon application, forming an effective barrier against fluid leakage and infection [12,13].

In this study, we developed and characterized corneal glue, a fast-curable polymer hydrogel composed of alginate, PVA, and CMC, for potential corneal defect repair. Formulations were optimized for rapid gelation, strong wet adhesion, and mechanical and optical properties suitable for ocular use. Cytocompatibility was evaluated using human corneal epithelial cells, and ex vivo testing on cadaveric human corneas assessed sealing performance relative to commercial cyanoacrylate and fibrin adhesives. We hypothesize that the PVA/SA/CMC hydrogel will exhibit significantly faster gelation than conventional fibrin adhesives, and maintain high cytocompatibility and support epithelial migration, and also achieve superior burst pressure and adhesive performance in ex vivo corneal models. Collectively, this work introduces corneal glue as a rapid, safe, and effective bioadhesive platform for corneal wound closure, offering a potential alternative to current adhesive and surgical approaches in ophthalmology.

## 2. Results and Discussion

The corneal glue (PVA/SA/CMC hydrogel) precursor was clear, homogeneous, and viscous, demonstrating excellent injectability and handling properties. Upon exposure to Ca^2+^ ions, the solution rapidly gelled, forming a stable three-dimensional network with high water content and mechanical strength, suitable for bioadhesive applications in corneal defect repair.

Hydrogel formation is driven by a combination of physical entanglement and ionic crosslinking. Hydrogen bonding and chain interactions among PVA, sodium alginate, and CMC create a physically entangled polymer network, while Ca^2+^ ions induce ionic crosslinking of alginate via coordination with guluronic acid residues, consistent with the classical egg-box model [14]. This dual mechanism ensures rapid gelation, robust tissue adhesion, and high water retention, offering mechanical stability superior to fibrin glue (tensile strength ~0.35 MPa vs. 0.12 MPa in the literature benchmarks) and comparable lap shear performance (~0.28 MPa vs. 0.10–0.15 MPa for similar hybrid hydrogels).

### 2.1. Characterization

**Figure 1:**
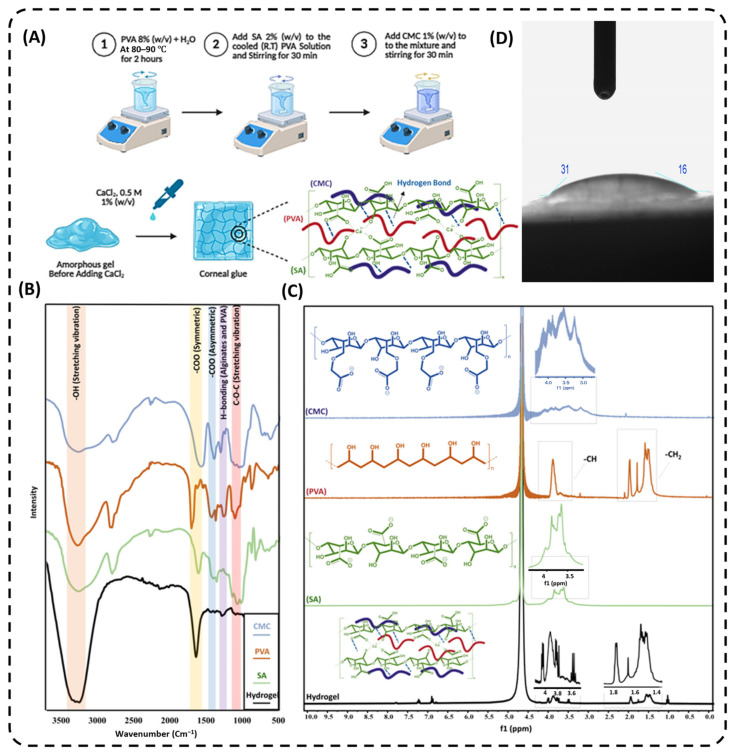
The corneal glue fabrication scheme, step by step (**A**), FT-IR spectra (**B**) and ^1^HNMR spectra (**C**) of the CMC, PVA, SA, and the final fabricated hydrogel (corneal glue). The analyzed contact angle image of the corneal glue (**D**).

As schematically depicted in Figure 1A, the corneal glue was fabricated using a controlled solution-blending approach combined with ionic crosslinking. The obtained corneal glue was subsequently characterized to evaluate its physicochemical properties, adhesive performance, and biocompatibility.

The FTIR spectra of the fabricated corneal glue confirmed the successful formation of a blended and ionically crosslinked polymeric network (Figure 1B). A broad absorption band was observed in the 3200–3500 cm^−1^ range, corresponding to hydroxyl (–OH) stretching vibrations [15]. In the corneal glue spectrum, this band appeared broadened and slightly shifted relative to the spectra of the individual polymers, indicating enhanced hydrogen-bonding interactions within the composite network. Characteristic peaks at approximately 1600–1650 cm^−1^ and 1400–1450 cm^−1^ were attributed to the asymmetric and symmetric stretching vibrations of carboxylate (–COO^−^) groups from alginate and carboxymethyl cellulose [16,17,18]. In the corneal glue spectrum, these peaks exhibited subtle shifts and changes in intensity compared to pure alginate, reflecting ionic interactions between the polymer chains during crosslinking.

Additionally, absorption bands in the 1000–1150 cm^−1^ region, corresponding to C–O–C and C–O stretching vibrations of polysaccharide backbones and PVA chains, were observed [19,20]. In the corneal glue spectrum, these bands appeared more integrated and slightly shifted, suggesting structural blending of the polymer components. No new characteristic peaks were detected, indicating that no covalent modifications occurred during hydrogel formation. Overall, the FTIR analysis demonstrates that corneal glue is composed of physically blended and ionically crosslinked polymers, with intermolecular interactions contributing to its network integrity and functionality.

FTIR analysis confirmed these interactions. The broadening and slight shifts in the –OH stretching band indicate enhanced intermolecular hydrogen bonding among the polymers, strengthening structural cohesion. Shifts in asymmetric and symmetric –COO^−^ bands verify the coordination of Ca^2+^ ions with guluronic acid blocks, supporting ionic crosslinking via the egg-box model. No new peaks were observed, confirming that the hydrogel network forms predominantly through physical entanglement and Ca^2+^-mediated ionic crosslinks rather than covalent bonding [21,22].

The ^1^H NMR spectra of corneal glue and its constituent polymers revealed characteristic signals corresponding to their structural features (Figure 1C). In CMC, the ring protons of the anhydroglucose units appeared in the 3.0–4.2 ppm region, while the methylene protons of the –CH_2_OCH_2_COO^−^ substituent were observed near 3.3–3.6 ppm, confirming successful carboxymethylation. PVA displayed methine (–CH–OH) protons around 3.8–4.0 ppm and methylene (–CH_2_–) protons between 1.3 and 1.6 ppm, with sharp peaks indicating high chain mobility. Alginate exhibited ring protons between 3.4 and 4.2 ppm and an anomeric proton near 4.7–5.0 ppm, reflecting differences between mannuronic and guluronic acid residues [20,23].

^1^H NMR spectroscopy further supports this mechanism. Peak broadening in the hydrogel indicates restricted molecular motion due to hydrogen bonding and polymer–polymer interactions, while subtle shifts in methylene and anomeric proton signals suggest effective Ca^2+^ coordination. These spectral changes collectively confirm the formation of a three-dimensional network with reduced chain mobility, consistent with a stable bioadhesive hydrogel.

In the corneal glue hydrogel, significant broadening of the 3.0–4.2 ppm signals were observed, corresponding to CMC and alginate protons, while the PVA methylene signals (1.3–1.6 ppm) became broader and slightly shifted. Minor shifts in the anomeric proton region were also noted, whereas no new resonances appeared. These results indicate that hydrogel formation in corneal glue occurs mainly via physical interactions and ionic crosslinking rather than covalent bonding. As shown in Figure 1D, the hydrogel surface exhibited low contact angles, approximately 31° and 16° at different regions of the droplet interface, indicating a highly hydrophilic surface. Pronounced droplet spreading and asymmetric reduction in the contact angle over time were observed, reflecting rapid water absorption and strong affinity between the hydrogel surface and the aqueous phase. These results confirm that the corneal glue possesses excellent wettability, which is favorable for interactions with biological tissues [24,25].

The corneal glue exhibited high surface hydrophilicity, as evidenced by low water contact angles. Abundant hydroxyl and carboxylate groups facilitate hydrogen bonding with water, promoting intimate contact with the moist corneal surface, enhancing interfacial adhesion, and enabling rapid conformal coverage of irregular wound geometries. This hydrophilic environment also supports Ca^2+^ ion diffusion, stabilizing ionic crosslinks and promoting fast curing. Such surface characteristics are critical for ophthalmic bioadhesives, as they enhance tissue integration and adhesion while maintaining a hydrated microenvironment conducive to healing [26,27,28].

### 2.2. Rheological Characterization

**Figure 2:**
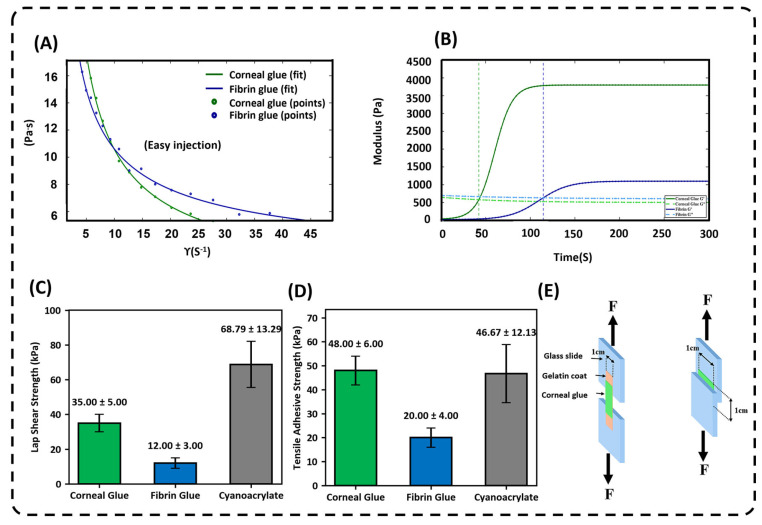
(**A**) Viscosity (Pa) as a function of shear rate for corneal glue and fibrin glue, demonstrating shear-thinning “easy injection” behavior. (**B**) Rheological time-sweep analysis showing the storage modulus (G’) and loss modulus (G”); the rapid crossover and plateau indicate efficient mechanical stabilization of the hydrogel. (**C**) Lap shear strength (kPa) and (**D**) tensile adhesive strength (kPa) of corneal glue compared to fibrin glue and cyanoacrylate, demonstrating high adhesive performance under different loading conditions. (**E**) Schematic illustration of the mechanical testing setups for evaluating shear and tensile adhesion on gelatin-coated substrates.

Steady shear rheology demonstrated that corneal glue exhibits pronounced shear-thinning behavior, characterized by a progressive decrease in viscosity with increasing shear rate. At low shear rates, the hydrogel maintains relatively high viscosity, promoting localized retention after application. Under elevated shear conditions, simulating extrusion through a fine-gauge cannula, viscosity decreases markedly, facilitating smooth and controlled delivery. This pseudoplastic response indicates that corneal glue can be easily injected while maintaining positional stability once shear forces are removed (Figure 2).

Oscillatory time-sweep analysis revealed rapid gelation following Ca^2+^ addition. Initially, the loss modulus (G″) exceeded the storage modulus (G′), indicating a predominantly viscous state. Within the first minute, G′ surpassed G″, confirming the formation of a solid-like elastic network [29,30]. Corneal glue reached a higher plateau storage modulus compared with fibrin glue, indicating greater elastic strength and improved structural integrity after curing. The sustained dominance of G′ over G″ throughout the plateau phase reflects a mechanically stable and deformation-resistant network [31,32].

Rheological analysis further confirms the formation of a structurally reinforced dual-network hydrogel. The marked shear-thinning behavior indicates that weak intermolecular interactions are temporarily disrupted under applied stress and rapidly reformed once stress is removed. This reversible response is characteristic of injectable polysaccharide-based hydrogels and is particularly valuable in ophthalmic applications, where smooth extrusion through a fine cannula must be combined with immediate positional stability after placement [33]. Time-sweep oscillatory measurements revealed a rapid crossover of the storage modulus (G′) over the loss modulus (G″) following Ca^2+^ addition, indicating fast sol–gel transition and early development of elastic behavior. The sustained dominance of G′ in the plateau phase confirms the establishment of a solid-like network capable of storing mechanical energy and resisting deformation under physiological stress. Similar rheological profiles with rapid G′/G″ crossover and elevated plateau modulus have been associated with enhanced burst resistance and mechanical durability in ionically crosslinked alginate systems. These rheological features correlate quantitatively with higher burst pressures (1.5–1.8× that of fibrin glue, *p* < 0.001) and mechanical durability compared with similar alginate-based adhesives reported in the literature [30]. Collectively, these findings support that Ca^2+^-mediated “egg-box” coordination, reinforced by hydrogen bonding and polymer chain entanglement, produces a cohesive three-dimensional structure with rapid gelation and improved viscoelastic stability.

**Figure 3:**
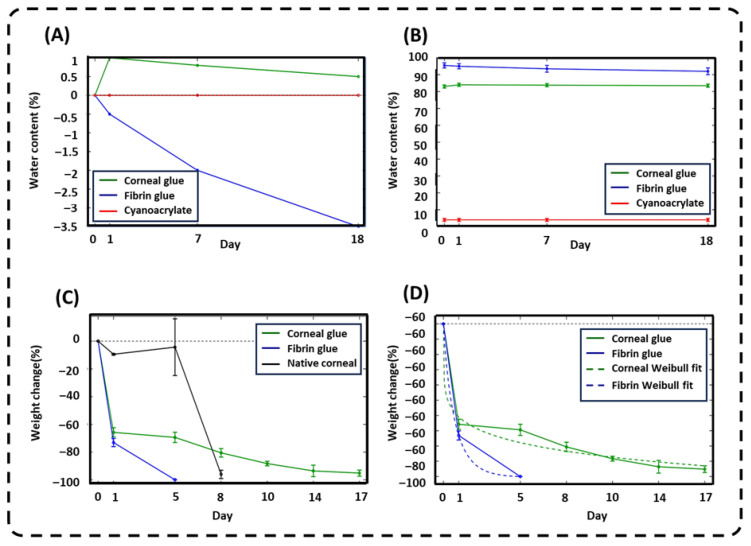
Physicochemical stability and degradation kinetics. (**A**,**B**) Relative and absolute water content (%) over 18 days, showing the high hydration stability of corneal glue versus fibrin glue and cyanoacrylate. (**C**) Enzymatic degradation profiles in collagenase; corneal glue exhibits gradual weight loss compared to the rapid degradation of fibrin glue and native cornea. (**D**) Experimental data with Weibull-type kinetic fits, highlighting the prolonged degradation “tail” of corneal glue over protein-based sealants.

Mechanical adhesion testing further confirmed the enhanced performance of corneal glue. In tensile testing, it exhibited significantly greater adhesive strength than fibrin glue, demonstrating improved resistance to separation forces. Similarly, lap shear testing showed that corneal glue withstood higher shear stresses before failure, indicating stronger interfacial bonding and cohesive network integrity. Although cyanoacrylate displayed high strength values, its mechanical response differed due to its rigid and brittle characteristics (Figure 3).

Mechanical adhesion tests further validate this structural enhancement. The notably higher tensile strength relative to fibrin glue indicates superior resistance to forces acting perpendicular to the adhesive interface, such as intraocular pressure. Likewise, increased lap shear strength reflects improved tolerance to tangential stress generated by blinking and tear flow. This enhanced performance is attributed to the hybrid network design, where ionic crosslinks provide immediate structural cohesion while polymer entanglement and secondary interactions improve toughness and stress distribution. In contrast, fibrin-based adhesives, which form through enzymatic polymerization, generally exhibit lower crosslink density and elastic modulus, limiting their mechanical resilience under continuous loading [34]. The simultaneous improvement in both tensile and shear strength highlights a balanced combination of interfacial bonding and internal cohesion: an essential requirement for stable corneal defect closure under complex, multidirectional physiological forces.

Overall, these rheological and mechanical properties suggest that corneal glue combines injectability, rapid solidification, and robust adhesive performance, making it well suited for ophthalmic applications requiring precise placement and durable tissue sealing.

### 2.3. Swelling Properties

Corneal glue maintained a high and stable water content throughout the entire incubation period. Water content remained approximately 83–84% from Day 0 through Day 18, indicating minimal net swelling in PBS. In comparison, fibrin glue exhibited a higher initial water content (95–96%) but gradually declined over time to 92–93% by Day 18. Cyanoacrylate showed negligible water content (3–4%) with no significant change over time.

When expressed as Δ water content relative to Day 0, corneal glue displayed only a small positive change (+1% at Day 1, decreasing to +0.5% by Day 18). Fibrin glue showed a progressive negative Δ water content (approximately −0.5% at Day 1 to 3 4% by Day 18), while cyanoacrylate remained near baseline (0%). These results indicate that corneal glue is highly hydrated yet dimensionally stable in physiological buffer. The stable water content over 3 weeks suggests that its ironic and physical network structure resists excessive expansion, which is crucial for corneal applications where swelling could distort curvature or impair optical performance.

Compared to fibrin glue, corneal glue has slightly lower overall water content, consistent with its higher elastic modulus and stronger adhesion observed in mechanical testing, reflecting a denser and less “dilute” polymer network. The minimal hydration of cyanoacrylate aligns with its rigid polymeric structure and clinical observations of poor compliance relative to soft tissues. Overall, the combination of high hydration and minimal swelling drift in corneal glue supports both biocompatible tissue contacts and stable defect coverage.

Although anterior curvature and surface roughness were not directly measured, the swelling and water content results demonstrate high hydration with minimal variation (83–84% over 18 days, <1% change), indicating strong resistance to volumetric expansion. This dimensional stability, together with the shear-thinning and conformal application behavior, suggests a low likelihood of swelling-induced geometric distortion of the corneal surface. Nevertheless, quantitative assessment of corneal topography, surface regularity, and refractive outcomes will require in vivo evaluation and is the focus of ongoing studies.

### 2.4. Enzymatic Degradation

In collagenase, fibrin glue degraded rapidly, reaching near-complete mass loss by approximately Day 5. Native corneal tissue showed limited weight loss at early time points but then underwent rapid degradation around Day 8. In contrast, corneal glue exhibited a more gradual degradation profile: weight loss reached approximately −65% by Day 1, remained around −70% by Day 5, and continued to decline progressively to −95% by Day 17.

**Figure 4:**
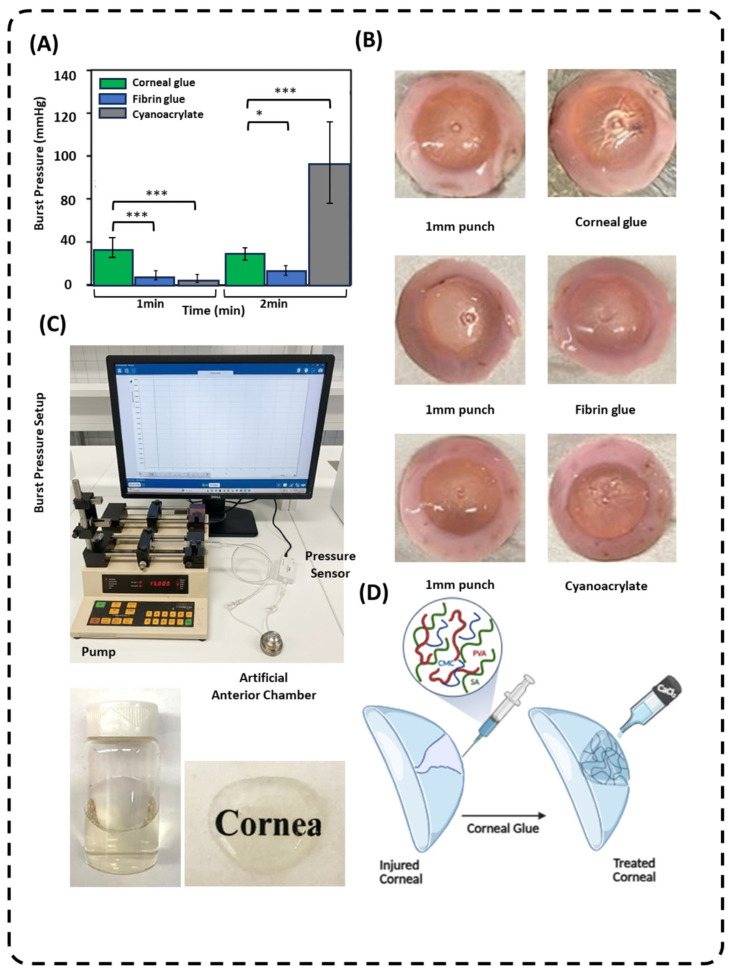
(**A**) Burst pressure of corneal glue, fibrin glue, and cyanoacrylate at 1 and 2 min post-application. Data are presented as mean ± SD (*n* = 10), with statistical significance indicated by stars relative to cyanoacrylate: * *p* < 0.05, *** *p* < 0.001. Corneal glue exhibited significantly higher burst pressure than fibrin glue at both time points (*** *p* < 0.001) and comparable or higher performance than cyanoacrylate at early time points (*** *p* < 0.001). (**B**) Representative photographs showing the application of adhesive materials to a cadaveric human corneal model with a 1 mm full-thickness wound, showing the cured state of the corneal glue at 1 min post-application. (**C**) Experimental setup for measuring burst pressure, featuring an artificial anterior chamber, pressure sensor, and infusion pump, (**D**) alongside a schematic illustration showing the application of the corneal glue to an injured cornea to achieve structural restoration and transparency.

The degradation curves were well described by a Weibull-type fit, indicating nonlinear degradation kinetics with a relatively slow “tail” for corneal glue compared to fibrin glue. These results suggest that corneal glue provides extended structural stability under enzymatic challenge, which is advantageous for maintaining mechanical support and corneal defect closure during early wound healing (Figure 4).

The enzymatic degradation profile further differentiates the hydrogel from protein-based sealants such as fibrin glue. Fibrin and other protein matrices are highly susceptible to proteolytic enzymes like collagenase, which cleave peptide bonds and destabilize networks. This susceptibility explains the rapid mass loss observed for fibrin glue and accelerated degradation of native corneal tissue under collagenase exposure [35]. In contrast, the corneal glue consists predominantly of non-protein polymers with ionic crosslinks that are not direct substrates for collagenase. Consequently, the slower mass loss observed during enzymatic challenge likely reflects gradual dissolution of soluble fractions and progressive weakening of the physically entangled network rather than enzymatic cleavage of primary polymer backbones. Clinically, this extended degradation profile is advantageous, as it maintains mechanical support and defect closure through critical early phases of wound healing without premature loss of adhesive integrity.

### 2.5. Burst Pressure Measurements

Burst pressure measurements were conducted at 1 and 2 min following wound repair to evaluate the sealing performance of corneal glue, fibrin glue, and cyanoacrylate. Corneal glue exhibited significantly higher burst pressure than fibrin glue at both time points, indicating superior early mechanical integrity and stable sealing.

To further elucidate the effect of polymer composition on sealing performance, different PVA/SA/CMC ratios were systematically evaluated in an ex vivo corneal model, and the results are summarized in Table 1. This design map highlights the trade-offs between formulation components and burst pressure. Increasing PVA content enhanced network stiffness but reduced flexibility at higher concentrations, while SA contributed to ionic crosslinking density and CMC improved conformability. The 8/2/1 formulation exhibited the highest burst pressure (35 ± 7 mmHg), indicating an optimal balance between mechanical strength and adaptability to the corneal surface.

Fibrin glue showed limited resistance throughout the testing period, whereas cyanoacrylate displayed low initial burst pressure followed by a marked increase of 2 min, consistent with rapid post-application hardening. Overall, these results demonstrate that corneal glue provides immediate and reliable wound closure, outperforming fibrin glue and offering more predictable early mechanical performance than cyanoacrylate. Data are presented as mean ± SD (*n* = 10), with statistical significance indicated by stars (* *p* < 0.05, ** *p* < 0.01) [9,30].

Burst pressure measurements further demonstrate the functional performance of corneal glue. Compared to fibrin glue and cyanoacrylate, the hydrogel exhibited significantly higher burst pressure within 1 and 2 min of application, indicating rapid mechanical stabilization and reliable wound sealing. The superior early burst pressure is consistent with the dual crosslinking mechanism, where physical entanglement provides immediate cohesive strength and Ca^2+^-mediated ionic crosslinks reinforce the network under applied pressure. Fibrin glue showed limited resistance at early time points, while cyanoacrylate exhibited delayed strengthening, reflecting its post-application hardening behavior. These results highlight that corneal glue provides predictable and immediate mechanical integrity, a key advantage in corneal defect repair where early leakage prevention is critical [35,36].

### 2.6. Effect of Hybrid Hydrogel on HCLE Viability and Proliferation

**Figure 5:**
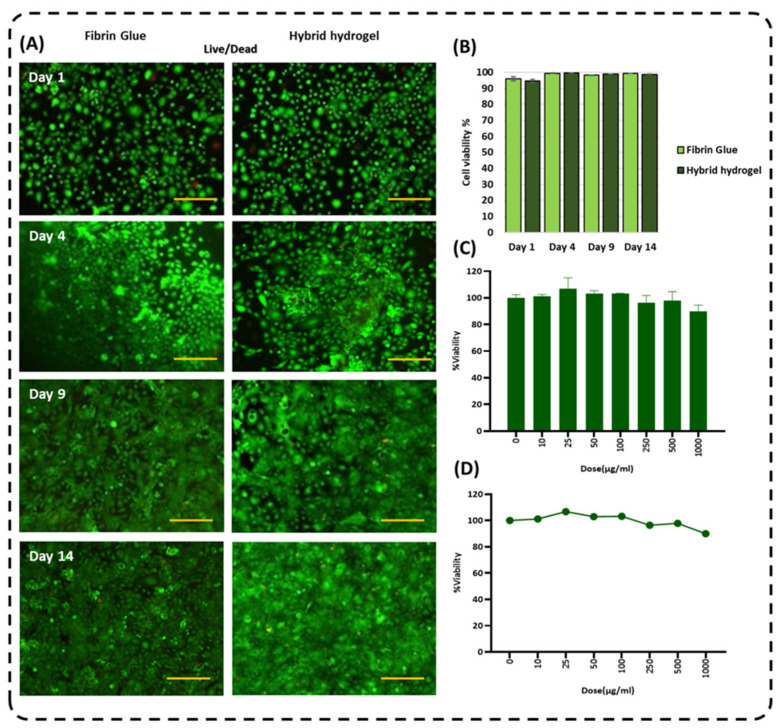
(**A**) Live/dead cell viability assays of HCLE were seeded on top of the hydrogel. Live cells emitted green and dead cells emitted red fluorescence (scale bar: 250 µm). (**B**) Percentage rates of cell viability for 14 days were calculated as proportion of total cell population. (**C**) The MTT assay of the cornea glue on epithelial normal cell line (MCF-10A) and (**D**) effect of increasing concentrations of the sample (0–1000 µg/mL) on cell viability.

The cytocompatibility of corneal glue was further evaluated in vitro using human corneal limbal epithelial cells (HCLE cells). Cells were seeded on the corneal glue surface, and their viability was monitored over 14 days using live/dead staining, with fibrin glue serving as a control. The live/dead assay demonstrated that both corneal glue and fibrin glue supported high HCLE viability throughout the culture period. Quantitative analysis showed that cell survival on corneal glue remained consistently high and comparable to that on fibrin glue at all time points (Figure 5).

Consistent with these results, HCLE cells seeded on corneal glue exhibited sustained adhesion, proliferation, and progressive surface coverage over the 14-day culture period. Overall, these findings suggest that corneal glue provides a supportive and cytocompatibility substrate for HCLE survival and growth, creating favorable conditions for corneal epithelial wound healing.

The MTT assay further confirmed the excellent biocompatibility of corneal glue. Cell viability remained above 95% at all time points and was close to or exceeded 100% relative to the control group, indicating negligible cytotoxicity (Figure 5). These results collectively demonstrate that corneal glue is well tolerated by HCLE cells and is suitable for in vitro corneal tissue applications.

### 2.7. In Vitro Wound Healing

At the initial time point (24 h), a well-defined and uniform scratch area was clearly visible, with sharp wound edges and a consistent cell-free gap, confirming the reproducibility of the injury.

Following incubation with corneal glue, progressive cell migration toward the wound region was observed. At intermediate time points, the scratch width decreased significantly, and cells at the wound edges displayed elongated and polarized morphologies, indicative of active migratory behavior. The wound margins became increasingly irregular over time, reflecting dynamic cytoskeletal remodeling and collective cell movement.

**Figure 6:**
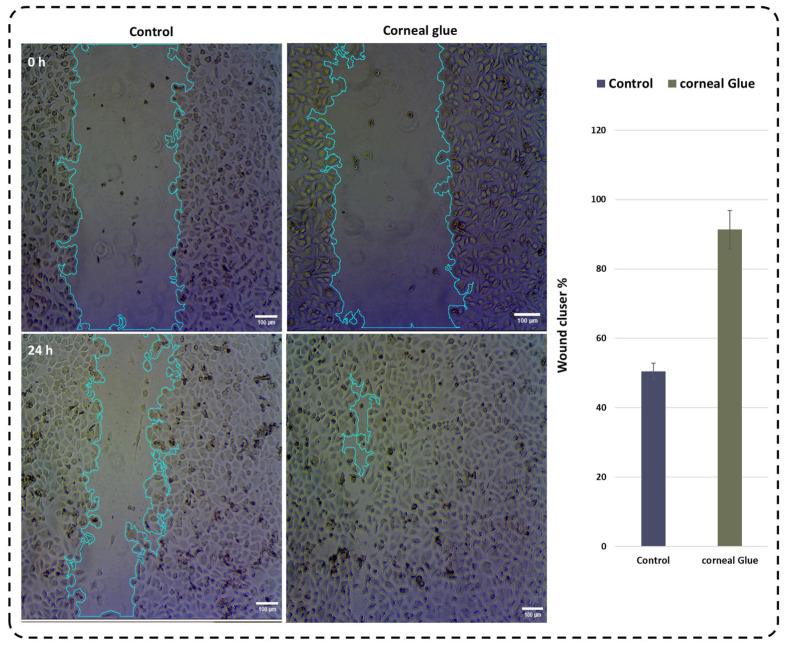
In vitro scratch wound healing assay on epithelial MCF10A cells. Blue lines indicate the wound area. The corneal glue group shows faster wound closure compared to control by 24 h. Scale bar: 100 µm.

By 24 h, near-complete closure of the scratch was achieved, with the gap almost entirely filled by newly migrated cells with ~92 ± 3% of the wound area filled by newly migrated cells, compared to the untreated control (51 ± 5%, *p* < 0.01) (Figure 6). The restored monolayer appeared dense and continuous, with minimal residual gaps. No evidence of cell detachment, rounding, or morphological abnormalities was observed in the corneal glue-treated groups, demonstrating the hydrogel’s supportive role in epithelial wound healing.

Biological studies demonstrated that corneal glue actively supports epithelial cell migration and wound closure. Scratch assays showed progressive narrowing of the cell-free area, with cells displaying elongated and polarized morphologies indicative of active migration. This behavior likely arises from the hydrogel’s hydrophilic surface, favorable chemical functionality, and cytocompatibility, collectively providing a permissive microenvironment for adhesion, proliferation, and directional migration key factors for corneal epithelial regeneration [37].

Overall, the corneal glue combines rapid gelation, mechanical stability, strong adhesion, and cytocompatibility, making it a promising candidate for corneal defect repair. Compared to fibrin-based sealants, it offers superior early mechanical integrity, while its mild ionic crosslinking avoids cytotoxic effects associated with covalent crosslinkers. These findings align with previous studies showing that hydrogels incorporating both physical entanglement and ionic crosslinking improve tissue adhesion and wound healing in ocular and soft tissue applications [38].

In summary, these findings demonstrate that the PVA/SA/CMC corneal glue exhibits dual-network cohesion via hydrogen bonding and Ca^2+^-mediated egg-box crosslinking, providing rapid gelation, enhanced mechanical performance, and active support for epithelial regeneration, highlighting its potential for clinical application in corneal defect repair.

## 3. Conclusions

Corneal glue demonstrates strong potential as a bioadhesive for corneal defect repair, combining rapid gelation, robust mechanical performance, and effective sealing capability through a dual mechanism of polymer entanglement and Ca^2+^-mediated ionic crosslinking. The material supports epithelial cell compatibility and maintains structural integrity during early-stage wound healing.

These findings highlight its promise as a translational candidate for ophthalmic applications. Future studies will focus on in vivo evaluation in relevant animal models to assess long-term safety, biocompatibility, and functional outcomes.

## 4. Materials and Methods

### 4.1. Hydrogel Fabrication

As Illustrated in Figure 1A, corneal glue was synthesized using a controlled solution-blending approach combined with ionic crosslinking, specifically engineered for rapid-curing bioadhesive applications in corneal defect repair. The method ensured uniform mixing of polyvinyl alcohol (PVA), sodium alginate (SA), and carboxymethyl cellulose (CMC), followed by crosslinking under mild conditions to achieve fast gelation, optimal mechanical strength, and strong tissue adhesion suitable for ocular use. An 8% (*w*/*v*) PVA solution was prepared by dissolving of deionized water at 80–90 °C under continuous stirring for 2 h until a clear, homogeneous solution was obtained. This elevated temperature disrupts the strong intermolecular hydrogen bonding within PVA chains, enabling uniform molecular dispersion, which is critical for achieving optical transparency and consistent mechanical properties required for corneal applications. The solution was then cooled to room temperature prior to further use. Sodium alginate 2% (*w*/*v*) was gradually added to the cooled PVA solution and stirred for 30 min to ensure uniform mixing. Subsequently, carboxymethyl cellulose (CMC), 1% (*w*/*v*) was incorporated into the polymer mixture and stirred for an additional 30 min to produce a homogeneous precursor solution.

### 4.2. Fourier Transform Infrared (FT-IR) Spectroscopy

FTIR spectroscopy was performed to characterize the chemical structure and intermolecular interactions of the individual polymers (PVA, SA, and CMC) as well as the fabricated corneal glue hydrogel. Spectra were recorded using a JASCO FT/IR-6600 spectrometer (JASCO Inc., Tokyo, Japan) across an appropriate scanning range to identify functional groups and examine peak broadening, intensity changes, and spectral shifts resulting from polymer blending and ionic crosslinking [39]. Analysis focused on key regions: the –OH stretching region (3200–3500 cm^−1^) to evaluate hydrogen bonding, the asymmetric and symmetric carboxylate (–COO^−^) stretching regions (1600–1650 cm^−1^ and 1400–1450 cm^−1^) to monitor ionic interactions, and the C–O–C/C–O stretching region (1000–1150 cm^−1^) to assess polymer backbone integrity [40].

### 4.3. Nuclear Magnetic Resonance (NMR) Spectroscopy

^1^H NMR spectra of the synthesized corneal glue hydrogel were recorded using a Bruker 500 MHz Advance instrument (Bruker Corporation, Billerica, MA, USA) with tetramethyl silane (TMS) as an internal standard and D_2_O as the deuterated solvent, and chemical shifts (δ) are reported in ppm. Signal multiplicities are indicated as singlet (s), doublet (d), triplet (t), quartet (q), or multiplet (m), following standard NMR reporting [41]. The NMR analysis focused on identifying characteristic polymer proton signals, detecting peak shifts and broadening associated with polymer blending and crosslinking, and evaluating molecular interactions within the corneal glue network, as commonly performed in hydrogel characterization studies using ^1^H NMR spectroscopy [42].

### 4.4. Contact Angle Measurement

The wettability and surface hydrophilicity of the corneal glue hydrogel were evaluated using static water contact angle measurements [43]. Water droplets were gently placed on the hydrogel surface, and contact angles were recorded at multiple points along the droplet interface to assess surface hydrophilicity. Measurements were performed in triplicate to ensure reproducibility, and representative images are shown in Figure 1D.

### 4.5. Rheological Characterization and Viscosity

The rheological and mechanical properties of corneal glue were evaluated to determine its suitability for corneal defects. Rheological measurements were performed using an Anton Paar MCR 302 rotational rheometer equipped (Anton Paar GmbH, Graz, Austria) with 25 mm parallel plate geometry under controlled temperature (25 °C). Steady shear viscosity was measured over a shear rate range of 0.1–100 s^−1^ [35]. Steady shear viscosity was measured over a range of shear rates relevant to extrusion through a 22-gauge cannula to assess flow behavior and handling characteristics during minimally invasive applications. Gelation kinetics and viscoelastic evolution were examined using oscillatory time-sweep measurements immediately after the addition of 0.5 M CaCl_2_, inducing ionic crosslinking of alginate chains. The storage modulus (G′) and loss modulus (G″) were recorded over time at a constant strain within the linear viscoelastic region, and gelation time was defined as the point where G′ surpassed G″, indicating the transition from a liquid-like precursor to a solid-like hydrogel network [44].

It was quantified using tensile and lap shear tests adapted from ASTM F2255 standards (Barroso, I.A., et al., Photocurable gelma adhesives for corneal perforations. Bioengineering, 2022. 9(2): p. 53.) [45]. Gelatin-coated glass slides (75 × 25 mm) were used as reproducible surrogate substrates to mimic a soft, hydrated interface. Slides were cleaned with ethanol, air-dried, and coated with a 5% gelatin solution. After drying under sterile conditions, pairs of gelatin-coated slides were overlapped with a defined bonded area (1 cm^2^) for tensile testing and a single-lap configuration (1 cm overlap) for shear testing. Corneal glue was applied evenly across the overlap region and allowed to cure at ambient temperature for 1 min before testing.

For tensile adhesion, bonded assemblies were mounted vertically in a universal testing machine, with the bonded area perpendicular to the loading axis. A uniaxial tensile force was applied at 1 mm·min^−1^ until failure, and adhesive strength was calculated as the peak load divided by the bonded area (MPa). Lap shear testing was performed with bonded slides oriented such that shear load was applied parallel to the interface. The maximum shear load at failure was normalized to the overlap area to determine apparent shear strength (MPa). All tests were conducted at ambient laboratory conditions, with at least five replicates per group to ensure statistical validity. Failure modes were documented for each specimen to distinguish adhesive from cohesive failure.

### 4.6. Ex Vivo Bioadhesion Strength Measurements

The bioadhesion strength of gel formulations, was evaluated using fresh cadaveric human corneas obtained from Eversight, Chicago, IL, USA [35]. Human corneas (average thickness ≈ 500 μm) were trephined to 10 mm in diameter and then bisected. Each corneal half was mounted onto a pre-cut contact lens holder to maintain native curvature. Approximately 2 mm of each corneal half was bonded to the contact lens holder with cyanoacrylate glue (Krazy Glue, Elmer’s Products Inc., Columbus, OH, USA). The holders were secured in the grips of a mechanical testing machine (225 lbs actuator with a 5 N load cell, Test Resources) and positioned with a 1 mm gap between the tissue edges.

The separated halves were joined using gels of varying degrees of functionalization (cured for 1 min with CaCl_2_), cyanoacrylate, or fibrin glue, applied over a bonding area of 3 mm in length and 0.5 mm in thickness using a 22-gauge angled cannula. For the fibrin control, curing was performed with cacl2 for 1 min. The tensile test was performed at a crosshead speed of 1 mm·min^−1^, and adhesion strength (MPa) was calculated by dividing the maximum recorded load (N) by the bonded surface area (3 mm × 0.5 mm). All samples (N = 6 per group) were tested to determine bioadhesion strength.

### 4.7. Ex Vivo Burst Pressure Measurements

The bioadhesive sealing performance of corneal glue formulations was evaluated using fresh human cadaveric corneas obtained from Eversight (Illinois, USA). Corneas were mounted in an artificial anterior chamber connected to a pressure sensor and an infusion pump aligned at the same height to maintain controlled intracameral pressure conditions, as previously described in corneal sealant evaluation studies [35]. Full-thickness corneal defects (1 mm diameter) were created using a biopsy punch under a baseline intracameral pressure of 18 mmHg.

Following defect creation, the wound surface was gently dried, and sealants were applied to the perforation using a 22-gauge cannula. Corneal glue formulations were crosslinked with CaCl_2_ and burst pressure measurements were performed at 1 and 2 min after crosslinker application to evaluate time-dependent adhesive maturation. Fibrin glue (activated with thrombin) and cyanoacrylate adhesive served as control groups.

Intracameral pressure was gradually increased by advancing a 20 mL syringe plunger connected to the chamber, while the infusion pump delivered fluid at a constant rate of 15 mL·h^−1^. Burst pressure was recorded in real time using SPARKvue software (version 4.12.4) and defined as the maximum pressure sustained before leakage occurred at the defect site [46]. Data were analyzed to compare the sealing capacity of corneal glue with commercial adhesive controls.

### 4.8. Enzymatic Degradation Assay

The enzymatic stability of corneal glue hydrogels was evaluated using a collagenase degradation assay, a widely used method for assessing biodegradability of hydrogel-based biomaterials. Fibrin glue and native human corneal tissue served as controls. Corneal glue samples were cast using a 10 mm silicone ring mold, dried, and weighed to determine their initial dry mass (*W*_0_). Each specimen was incubated in 200 µL of bacterial collagenase type I (Sigma-Aldrich, St. Louis, MO, USA) at a concentration of 5 U·mL^−1^ in 50 mM TES buffer containing 0.36 mM CaCl_2_ at 37 °C to simulate physiological conditions [38].

Samples were retrieved at predetermined time points (Days 0, 1, 5, 8, 11, 14, and 17), gently rinsed with distilled water to remove residual enzyme, and vacuum-dried to a constant mass. The remaining dry weight (*W_t_*) was recorded, and percentage weight loss was calculated using the following equation:

$$\mathrm{Percent}\;\mathrm{Weight}\;\mathrm{Loss}=(\frac{W_{0}-W_{t}}{W_{0}})\times 100$$

where $W_{0}$ is the initial dry weight and $W_{t}$ is the dry weight after enzymatic exposure at each time point. This procedure enabled quantitative monitoring of hydrogel degradation over the six-week period.

### 4.9. Swelling Ratio and Water Content Analysis

The swelling behavior and water content of corneal glue hydrogels were evaluated to assess their hydration capacity and structural stability under physiological conditions, as commonly performed for hydrogel [47]. Disk-shaped constructs (7 mm diameter, 520 µm thickness) were fabricated by dispensing 80 µL of each hydrogel formulation into polytetrafluoroethylene (PTFE) molds. Fibrin glue, including its fibrinogen and thrombin components, was prepared and used as a comparative control. The fibrin constructs were molded in the same geometry and incubated at 37 °C for 30 min to ensure complete polymerization.

Cadaveric human corneas obtained from Eversight (Illinois, USA; average thickness 500–550 µm) were trephined using a 7 mm biopsy punch and included as a biological reference. All samples were briefly rinsed with phosphate-buffered saline (PBS), gently blotted dry, and weighed to determine their initial weight (*W*_0_). Samples were then incubated in 500 µL PBS at 37 °C, and their weight was recorded on Day 1 (*W*_1_), Day 7 (*W*_7_), and Day 18 (*W*_18_) following gentle surface drying with KIMTECH Kimwipes. At the conclusion of the incubation period, all samples were dried at 90 °C for 3 h to determine their dry weight (*DW*). Water content (%) at each time point was calculated using the following equation:

$$\mathrm{Water}\;\mathrm{Content}\;(\%)=\frac{\left(W_{t}-DW\right)}{W_{t}}\times 100$$

where *W_t_* represents the swollen weight at each designated time point and DW represents the final dry weight. This analysis enabled quantitative comparison of hydration capacity between corneal glue, fibrin control, and native corneal tissue.

### 4.10. Viability, Proliferation, and Migration on the Hydrogel Surface

Live/Dead Assay (HCLE Cells)

To mimic the physiological corneal microenvironment, 50 μL of corneal glue precursor solution was deposited into the center of each well of an 8-well chamber slide. Corneal glue was crosslinked with calcium chloride to induce ionic gelation, while fibrin glue was polymerized with thrombin according to the manufacturer’s protocol, yielding constructs approximately 500 μm in thickness. Human corneal epithelial cells (HCLE cells), with an immortalized line at passage 5, were seeded onto the surface of corneal glue at a density of 5 × 10^4^ cells per well. Cultures were maintained for up to 15 days at 37 °C in a humidified incubator with 5% CO_2_, consistent with standard in vitro corneal epithelial culture conditions.

On Days 1, 4, 9, and 15 post-seeding, cell morphology, proliferation, and migration on the corneal glue surface were evaluated using phase-contrast microscopy. Cell viability was assessed using a live/dead assay based on Calcein-AM (live cells) and Propidium Iodide (PI, dead cells), following established fluorescence staining protocols for hydrogel cytocompatibility assessment. Fluorescent imaging was performed using an EVOS fluorescence microscope, and quantitative analysis of cell coverage, migration distance, and proliferation rate was conducted using ImageJ software (ImageJ 1.53) [48]. This assay provides a comprehensive evaluation of epithelial cell responses to corneal glue, including survival, growth, and surface migration, thereby modeling early cellular events associated with corneal epithelial wound healing.

Indirect cytotoxicity of corneal glue was evaluated using an MTT assay on normal epithelial MCF10 cells, following established hydrogel cytocompatibility protocols. Cells were exposed to corneal glue extracts at concentrations ranging from 0 to 1000 μg/mL for a defined incubation period [49]. After exposure, cell viability was determined by measuring metabolic activity using the standard MTT protocol. Absorbance values were recorded spectrophotometrically, and results were expressed as percentage viability relative to untreated control cells. This assay provided quantitative assessment of the cytotoxic potential of corneal glue extracts, supporting its safety for ocular applications.

### 4.11. In Vitro Wound Healing (Scratch Assay)

An in vitro scratch (wound-healing) assay was performed to evaluate the effect of corneal glue on epithelial cell migration and wound closure, following previously reported hydrogel cytocompatibility and migration studies [50]. Confluent epithelial cell monolayers were mechanically injured by creating a uniform scratch using a sterile pipette tip to generate a reproducible wound. Detached cells were removed by gentle washing, and fresh culture medium containing corneal glue formulation was added.

Cell migration into the wound area and the extent of wound closure were monitored at predetermined time points using phase-contrast microscopy. Images were captured to assess changes in wound width and cellular morphology during the healing process. Quantitative analysis of wound closure was performed using ImageJ to measure the reduction in wound area over time. This assay provides a functional evaluation of corneal glue’s effect on epithelial cell motility and simulates early events in corneal epithelial repair.

### 4.12. Statistical Analysis

All data are presented as mean ± standard deviation (SD). Statistical analyses were performed using GraphPad Prism (version 10.3.0; GraphPad Software, San Diego, CA, USA). Differences between two groups were assessed using the t test, while comparisons between multiple groups were performed using one-way ANOVA followed by Tukey’s post hoc test. A *p* value of less than 0.05 was considered statistically significant.

## Figures and Tables

**Table 1:** Effect of PVA/SA/CMC ratios on burst pressure in an ex vivo corneal model.

Formulation (% *w*/*v*)	PVA	SA	CMC	Burst Pressure (mmHg)
7/3/1	7	3	1	18 ± 3
8/2/1 (Chosen)	8	2	1	35 ± 7
10/1/1	10	1	0.5	23 ± 4
8/3/0.5	8	3	0.5	16 ± 4
9/2/0	9	2	0	4 ± 2

## Data Availability

The original contributions presented in this study are included in the article. Further inquiries can be directed to the corresponding authors.
